# Aquatic Ecosystem Deterioration and Its Potential Drivers in a Floodplain Lake in the Lower Yellow River Area, North China

**DOI:** 10.1002/ece3.72946

**Published:** 2026-01-11

**Authors:** Yingying Chen, Yanyu Ji, Qinghui Zhang, Yunwei Zhang, Shi‐Yong Yu, Shiyue Chen

**Affiliations:** ^1^ School of Geography, Geomatics, and Planning Jiangsu Normal University Xuzhou Jiangsu China; ^2^ State Key Laboratory of Estuarine and Coastal Research East China Normal University Shanghai China; ^3^ College of Geography and Environment Shandong Normal University Ji'nan Shandong China

**Keywords:** aquatic vegetation, community structure, Dongping Lake, vegetation decline, water quality

## Abstract

Lake Dongping is a floodplain lake in the lower Yellow River area experiencing ecosystem deterioration. Understanding the structure and succession of its aquatic communities is essential for ecological restoration. However, due to lack of long‐term monitoring data, the onset and causes of ecosystem degradation in this lake are unclear. This study employed Landsat imagery and Support Vector Machine (SVM) to extract spring and summer aquatic vegetation distribution in Dongping Lake, combined with field survey data and environmental factors to analyze its succession over the past 40 years. Redundancy Analysis (RDA) was then used to investigate the factors driving aquatic ecosystem deterioration. Results indicate that since 1979, aquatic vegetation in Dongping Lake has generally declined, with species diversity decreasing from 40 to 6 species and community structure becoming increasingly simplified. Pollution‐sensitive vegetation has experienced substantial reductions in both distribution area and biomass. The period around 2000 marked a critical transition when pollution‐tolerant *Potamogeton crispus* emerged as a dominant monoculture while original dominant species continued to decline. Water quality and water levels were identified as primary drivers of aquatic vegetation succession, with poor water quality and high water levels being key factors in community degradation. Meteorological conditions also contributed to aquatic vegetation changes. Human activities including aquaculture and the South‐to‐North Water Diversion Project mainly influenced aquatic vegetation succession indirectly by altering water quality and water level conditions. Our results provide valuable insights for assessing ecosystem health and offer potential management strategies for future ecological restoration of Dongping Lake.

## Graphical abstract

This study employed Landsat imagery and Support Vector Machine to extract spring and summer aquatic vegetation distribution in Dongping Lake, combined with field survey data and environmental factors to analyze its succession over the past 40 years. Redundancy Analysis was then used to investigate the factors driving aquatic ecosystem deterioration.

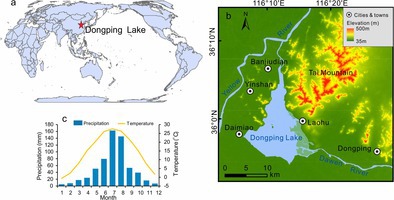

## 1 Introduction

Lakes, as integral components of the Earth's system, play vital roles in water conservation, environmental purification, climate regulation, and the maintenance of biodiversity. As a key primary producer in lake ecosystems, aquatic vegetation serves as a crucial research focus due to its significant contributions to stabilizing ecosystem structure and function (Heino et al. 2021). On one hand, it provides food sources and habitats for other organisms while suppressing the growth of planktonic algae. On the other hand, through physiological processes or by reducing water turbulence, aquatic plants effectively decrease nutrient and pollutant concentrations in water bodies (Dong et al. 2018). Over the past one to two centuries, under the compounded impacts of climate change and human activities, lake ecosystems worldwide have experienced severe degradation (Sand‐Jensen et al. 2000; Smol 2019; Huang et al. 2022; Suba et al. 2025; Zhang, Wu, and Yang 2025). Notably, 65.2% of lakes have seen significant declines in aquatic vegetation, with submerged macrophytes accounting for 65.3% of these losses in coverage (or area) (Zhang et al. 2017).

The decline of aquatic vegetation has driven many shallow lakes to shift from clear “macrophyte‐dominated” states to turbid “algae‐dominated” states (Ho et al. 2019; Huang et al. 2021), leading to the collapse of key ecosystem services and severely constraining socioeconomic development in watersheds (Lin et al. 2019; Luo et al. 2025). In light of these challenges, restoring lakes from turbid to clear states remains a central goal of lake management, with the effective recovery of aquatic vegetation communities being a critical step. A deep understanding of the historical processes, patterns, and mechanisms underlying aquatic vegetation degradation is essential for establishing restoration targets and pathways, providing crucial insights for ecological rehabilitation and management.

Lake monitoring programs in China are relatively short in duration, typically initiated only after the emergence of ecological or environmental risks in lake systems. Traditional field surveys, while capable of accurately identifying detailed species and community compositions, are limited in spatial coverage and require significant time and labor (Huang et al. 2021). Remote sensing technology, with its broad spatial coverage, temporal continuity, and high precision, has become a key supplementary tool for lake ecological and environmental monitoring. However, its effectiveness can be constrained by factors such as weather conditions and water transparency (Liang et al. 2019; Zhang et al. 2019). Paleolimnological approaches, utilizing indicators such as pollen and plant macrofossils preserved in lake sediments, can provide long‐term, continuous records of aquatic vegetation community dynamics. Nevertheless, uncertainties may arise due to interspecific differences in sediment deposition processes, transport patterns, and productivity (Dong et al. 2018). Therefore, to reconstruct the historical dynamics of aquatic vegetation more accurately and comprehensively, it is advisable to integrate multiple research methods to facilitate cross‐validation and mutual supplementation. This integrated approach can provide more robust and holistic insights into the evolution of aquatic vegetation communities.

Dongping Lake, a shallow freshwater ecosystem in the lower Yellow River area and a critical reservoir of the East Route of China's South‐to‐North Water Diversion Project (SNWDP), holds strategic significance for national water security. This dual role as both an ecologically sensitive wetland and a critical hydraulic infrastructure node has garnered significant scientific attention. To date, multidisciplinary investigations have systematically examined its hydrochemistry, aquatic biodiversity, and sediment dynamics (Chen et al. 2023, 2024; Li et al. 2018; Yu et al. 2020; Zhang, Chen, et al. 2025). Since the 1980s, it has experienced remarkable ecological transformations over four decades, transitioning through distinct eutrophication stages accompanied by progressive aquatic vegetation loss (Wang 1984; Yu et al. 2005; Tian et al. 2018). Yet, the critical drivers remain unresolved. Current studies predominantly focus on recent ecological challenges, lacking systematic deconstruction of four‐decade vegetation succession, thereby hindering targeted restoration strategies. To address this, we synergize multi‐temporal Landsat imagery (1984–2023) processed via Support Vector Machine (SVM) classification with in situ ecological surveys. This integrated approach enables systematic reconstruction of spring–summer vegetation dynamics at annual resolution, while redundancy analysis (RDA) of coupled environmental datasets elucidates dominant drivers of ecological succession. Our study aims to (1) quantify historical trajectories of aquatic vegetation loss, (2) identify primary forcing mechanisms across temporal scales, and (3) propose adaptive management strategies grounded in the lake's current ecological status. The findings provide potential management strategies for future ecological restoration of Dongping Lake.

## 2 Study Area

**FIGURE 1:**
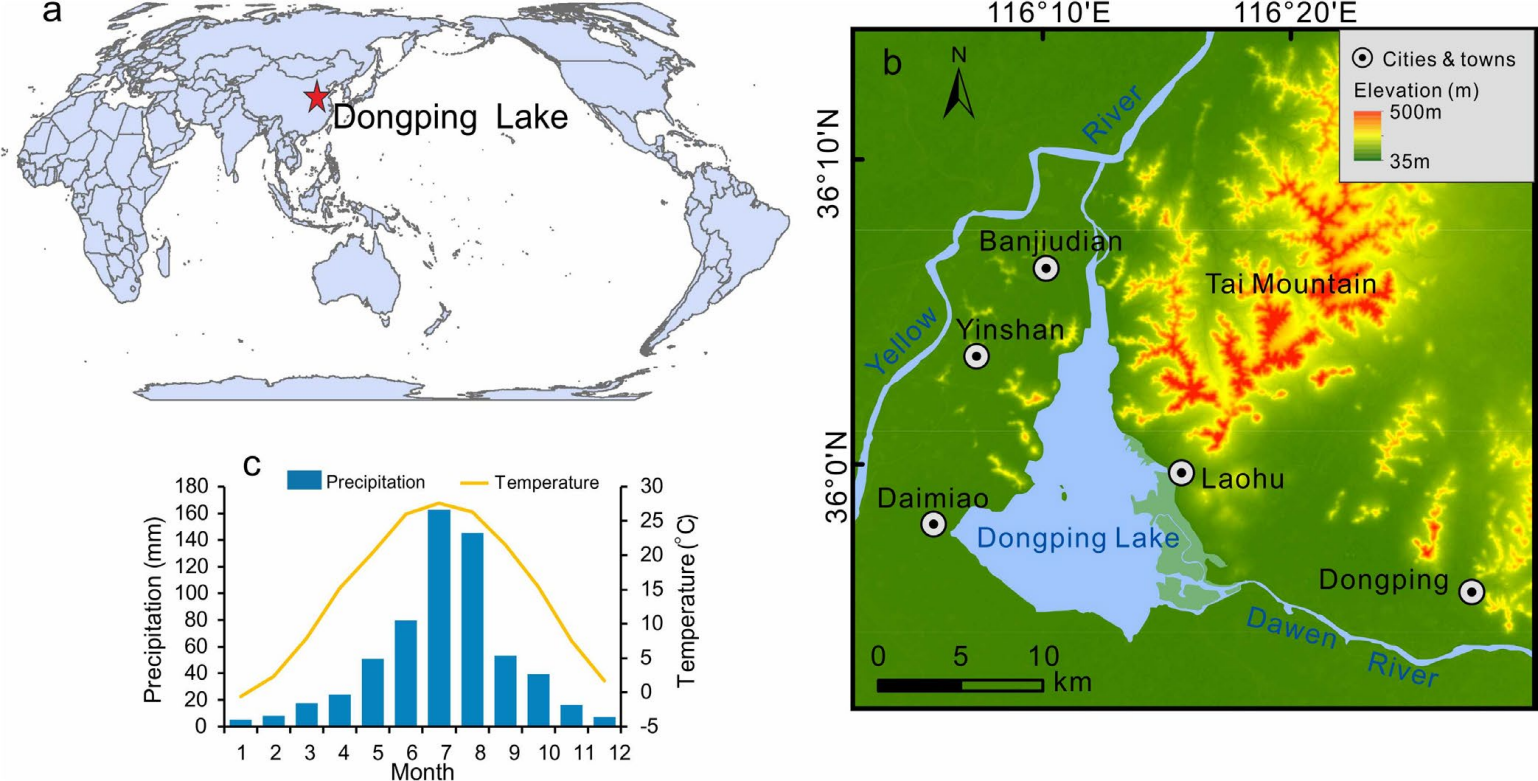
Map of the study area (a: Location of the Dongping Lake (red star), b: Geomorphological features of Dongping Lake, c: Climatology of Dongping County (1978–2003 ce)).

Dongping Lake (N 35°30′–36°20′, E 116°00′–116°30′) is a floodplain lake in Dongping County, Shandong Province, North China (Figure 1a). With a total area of 627 km^2^ and an average depth of 2–4 m, the lake maintains a perennial water surface of 124 km^2^ and a storage capacity of 4.0 × 10^9^ m^3^ (Dongping Lake Administration 2014). It gradually took its current shape after 1855. After the Yellow River reverted to its old course in Shandong in 1947, Dongping Lake became a natural flood‐storage area for the Yellow River and the Dawen River. Additionally, it serves as a critical regulatory reservoir for the East Route of China's SNWDP, ensuring water redistribution and quality control for transferred Yangtze River water.

It hydrologically connects to the Dawen River in the east and the Yellow River in the north (Figure 1b), functioning primarily as a natural flood diversion basin during extreme Yellow River flood events. The history of Dongping Lake included significant changes due to frequent overflows and course changes of the Yellow River (Dongping Lake Administration 2014). The region experiences a warm‐temperate continental semi‐humid monsoon climate, characterized by distinct seasonal variability (Figure 1c). Mean annual temperature ranges from −0.6°C in January to 27.6°C in July, with 641 mm of annual precipitation concentrated predominantly in July–August (52% of yearly total).

## 3 Materials and Methods

### 3.1 Data Collection

This study utilized Landsat series satellite imagery data obtained from the Google Earth Engine (GEE) platform as the remote sensing data source for aquatic vegetation classification and extraction. To ensure spectral consistency and minimize potential impacts from sensor differences, only data from Landsat 5 and Landsat 8 satellites were employed. Using the GEE platform, 185 cloud‐minimized images covering the study area during the peak aquatic vegetation season (April to August) were selected. After radiometric calibration, the images were composited into raster data containing five spectral bands: Blue (B), Green (G), Red (R), Near Infrared (NIR), and Shortwave Infrared (SWIR), in accordance with the specific needs of this study.

Environmental factor data were collected to investigate potential natural and anthropogenic drivers of aquatic vegetation evolution in Dongping Lake. Monthly water level data from 1991 to 2005 were obtained from the Dongping Lake Administration (2014), while data from 2006 to 2022 were acquired from the Yellow River Water Conservancy Commission of the Ministry of Water Resources website (http://www.yrcc.gov.cn). Monthly mean temperature data from 1984 to 2018 were extracted from the China Regional Surface Meteorological Elements Driving Dataset provided by the National Tibetan Plateau Data Center (He et al. 2020; Yang et al. 2010, 2019). Temperature data from 2019 onward were sourced from the Hefeng Weather website (https://www.qweather.com). To ensure continuity between datasets, temperature differences between HefengWeather data and the China Regional Surface Meteorological Elements Driving Dataset for 2017–2018 were calculated and used to adjust post‐2019 data. Results showed temperature discrepancies generally within 1°C, which were negligible for the study's requirements. Monthly mean wind speed and downward shortwave radiation data (hereafter referred to as “solar radiation”) were obtained from the NASA Prediction of Worldwide Energy Resources project (https://power.larc.nasa.gov).

Water quality parameters (Total Phosphorus (TP), Total Nitrogen (TN), Permanganate Index (COD_Mn_), Dichromate Index (COD_Cr_)) and eutrophication levels since 1990 were compiled from previous studies (Li et al. 2018; Liang et al. 2019) and the China Ecological Environment Status Bulletin published by the Ministry of Ecology and Environment (https://www.mee.gov.cn).

This comprehensive dataset enables systematic analysis of hydrological, climatic, and anthropogenic influences on aquatic vegetation dynamics in Dongping Lake.

### 3.2 Determination of Aquatic Vegetation Classification Criteria

Based on life forms, aquatic plants are categorized into emergent, floating‐leaved, free‐drifting, and submerged plants (Zhou et al. 2023). Emergent plants are rooted in sediment with their lower stems or bases submerged in water, while stems and leaves are exposed to air. Floating‐leaved plants are anchored to the substrate, with only their leaves or parts of their leaves floating on the water surface for photosynthesis. Free‐drifting plants remain entirely suspended on the water surface, with their roots submerged but unattached to the substrate. Submerged plants spend most of their life cycle fully underwater, with their bodies entirely submerged and rooted in the substrate. However, free‐drifting plants are typically excluded as a distinct category in remote sensing‐based aquatic vegetation studies (Luo et al. 2014). Consequently, this study incorporates free‐drifting plants when analyzing aquatic vegetation community dynamics using field data but focuses solely on submerged, floating‐leaved, and emergent vegetation for remote sensing extraction.

### 3.3 Aquatic Vegetation Extraction

**FIGURE 2:**
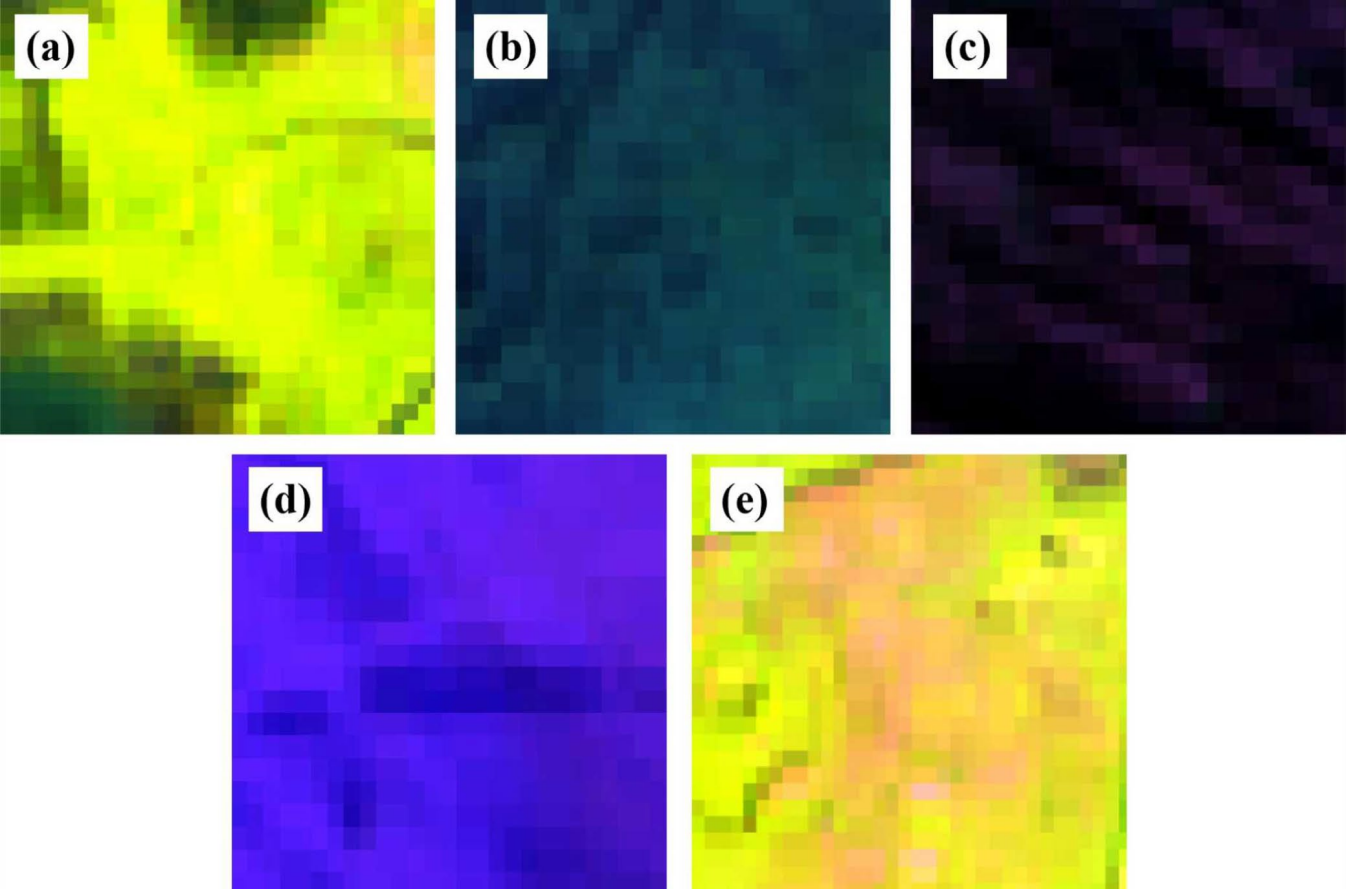
Different land covers in false‐color images (a–e correspond to emergent vegetation, floating‐leaf vegetation, submerged vegetation, open water, and bare land, respectively).

Selecting appropriate band combinations can visually highlight effective information from different spectral bands in remote sensing imagery, aiding in the visual identification of aquatic vegetation. Research by Luo et al. (2014) in Lake Taihu demonstrated that in standard false‐color composites (using near‐infrared, red, and green bands), emergent vegetation appears bright red, floating‐leaved vegetation as pink, submerged vegetation as dark green to deep gray, and open water as bright cyan. Building on prior experience and field survey data from Dongping Lake, this study conducted multiple trials and identified that a false‐color composite combining shortwave infrared, near‐infrared, and red bands (SWIR‐NIR‐R) effectively distinguishes aquatic vegetation in Dongping Lake. With this band combination, emergent vegetation appears bright yellow or yellowish‐green, floating‐leaved vegetation as darker green, submerged vegetation as deep blue to dark gray, open water as vivid blue or purple, and bare land as bright pink or light gray (Figure 2).

In ENVI 5.6 software, visual interpretation of land cover distribution in the study area was conducted primarily using SWIR‐NIR‐R false‐color composite imagery, supplemented by standard false‐color and true‐color imagery. Classification samples for open water, submerged vegetation, floating‐leaved vegetation, emergent vegetation, and bare land were established in each scene. Classification was performed using a Support Vector Machine (SVM) tool. To account for the phenological dynamics of aquatic vegetation in Dongping Lake, only spring imagery (flourishing period of *Potamogeton crispus* ) and summer imagery (flourishing period of other aquatic vegetation) were selected for classification. Temporal sequences were established separately, resulting in a total of 38 classified scenes: 23 spring scenes acquired between April 22 and May 14, and 15 summer scenes acquired between July 12 and August 30 (Table 1).

**TABLE 1:** Image types and acquisition times.

Year	Image type	Spring image acquisition time	Summer image acquisition time
1985	Landsat5 TM	04.22	—
1987	Landsat5 TM	05.14	07.17
1988	Landsat5 TM	05.07	08.04
1989	Landsat5 TM	—	08.14
1991	Landsat5 TM	05.09	—
1992	Landsat5 TM	05.02	07.30
1993	Landsat5 TM	05.14	—
1995	Landsat5 TM	05.04	—
1996	Landsat5 TM	—	08.17
1997	Landsat5 TM	05.09	07.12
1998	Landsat5 TM	05.03	—
1999	Landsat5 TM	05.06	—
2000	Landsat5 TM	05.01	08.21
2003	Landsat5 TM	05.10	—
2004	Landsat5 TM	—	07.22
2005	Landsat5 TM	05.06	—
2006	Landsat5 TM	05.02	—
2009	Landsat5 TM	—	08.30
2010	Landsat5 TM	04.27	—
2011	Landsat5 TM	—	07.26
2013	Landsat8 OLI	05.12	—
2014	Landsat8 OLI	04.29	08.03
2015	Landsat8 OLI	04.25	—
2016	Landsat8 OLI	05.04	—
2017	Landsat8 OLI	05.07	—
2018	Landsat8 OLI	05.03	07.22
2019	Landsat8 OLI	—	08.17
2020	Landsat8 OLI	04.29	08.28
2022	Landsat8 OLI	05.05	07.24

During the classification process, spectral overlap between submerged and floating‐leaved vegetation was observed, particularly in dense submerged vegetation patches where reflectance characteristics closely resembled those of floating‐leaved vegetation. Field surveys have also documented that floating‐leaved plants often coexist with other aquatic species (Yu et al. 2005). Consequently, submerged and floating‐leaved vegetation classes were merged post‐classification, resulting in four final classes: open water, submerged/floating‐leaved vegetation, emergent vegetation, and bare land.

The classified raster data were reprojected to the Universal Transverse Mercator (UTM) coordinate system using ArcGIS 10.8, and the areal extent of each land cover type was quantified using MATLAB software.

### 3.4 Redundancy Analysis

Redundancy analysis (RDA) quantitatively evaluates the effects of multiple explanatory variables on response variables and calculates their variance contribution rates (Birks 1998). This study employed RDA to explore relationships between aquatic vegetation area in Dongping Lake and environmental factors. Due to the qualitative nature of some environmental data (e.g., eutrophication levels) or limited temporal resolution (e.g., water quality metrics such as TN), only hydrological (water level) and meteorological factors were included in the RDA. In Canoco 5.0 software, using the spring and summer aquatic vegetation area of Dongping Lake as response variables and water level and meteorological indicators as explanatory variables, RDA was employed to investigate the quantitative relationships between aquatic vegetation area (of different seasons and types) and environmental factors. Explanatory variables are listed in Table 2, with the top five contributors based on variance contribution rates retained for final analysis.

**TABLE 2:** Environmental factors included in the RDA analysis.

Environmental factor	Aquatic vegetation growth stage
Average water level from May to August of the previous year	Growth period of the previous year
Average solar radiation intensity from May to August of the previous year	
Average temperature from May to August of the previous year	
Average temperature from September to October of the previous year	Seed dispersion period of the previous year
Average water level from September to October of the previous year	
Average water level from November of the previous year to February of the current year	Overwintering period of aquatic vegetation
Average water level from March to April of the current year	Spring aquatic vegetation growing season
Average temperature from March to April of the current year	
Average wind speed from March to April of the current year	
Average solar radiation intensity from March to April of the current year	
Average water level from March to July of the current year	Summer aquatic vegetation growing season
Average temperature from March to July of the current year	
Average wind speed from March to July of the current year	
Average radiation intensity from March to July of the current year	

## 4 Results and Discussion

### 4.1 Trends in the Succession of Aquatic Macrophytes Community

**FIGURE 3:**
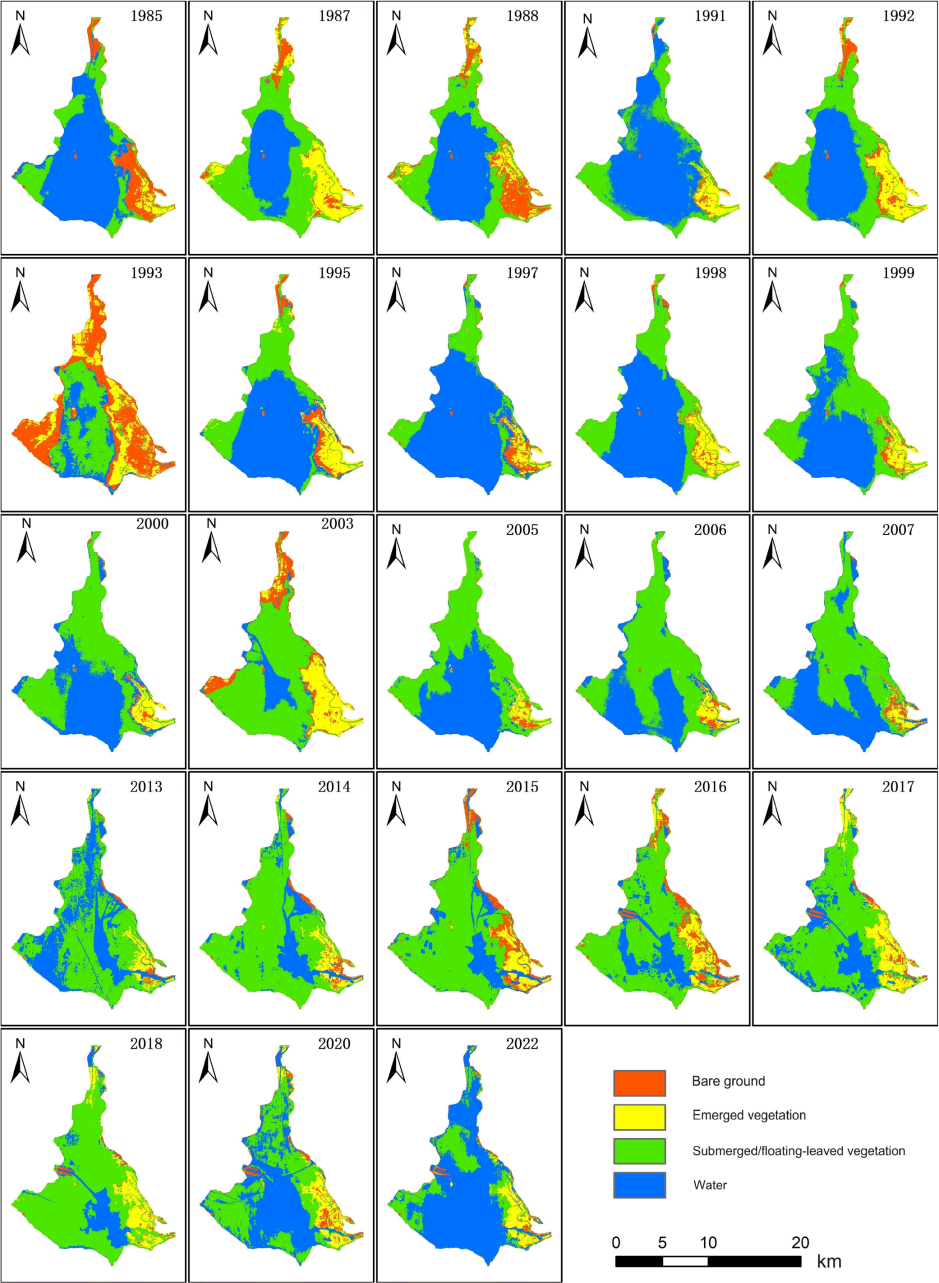
Distribution of aquatic vegetation in Dongping Lake during the spring.

**FIGURE 4:**
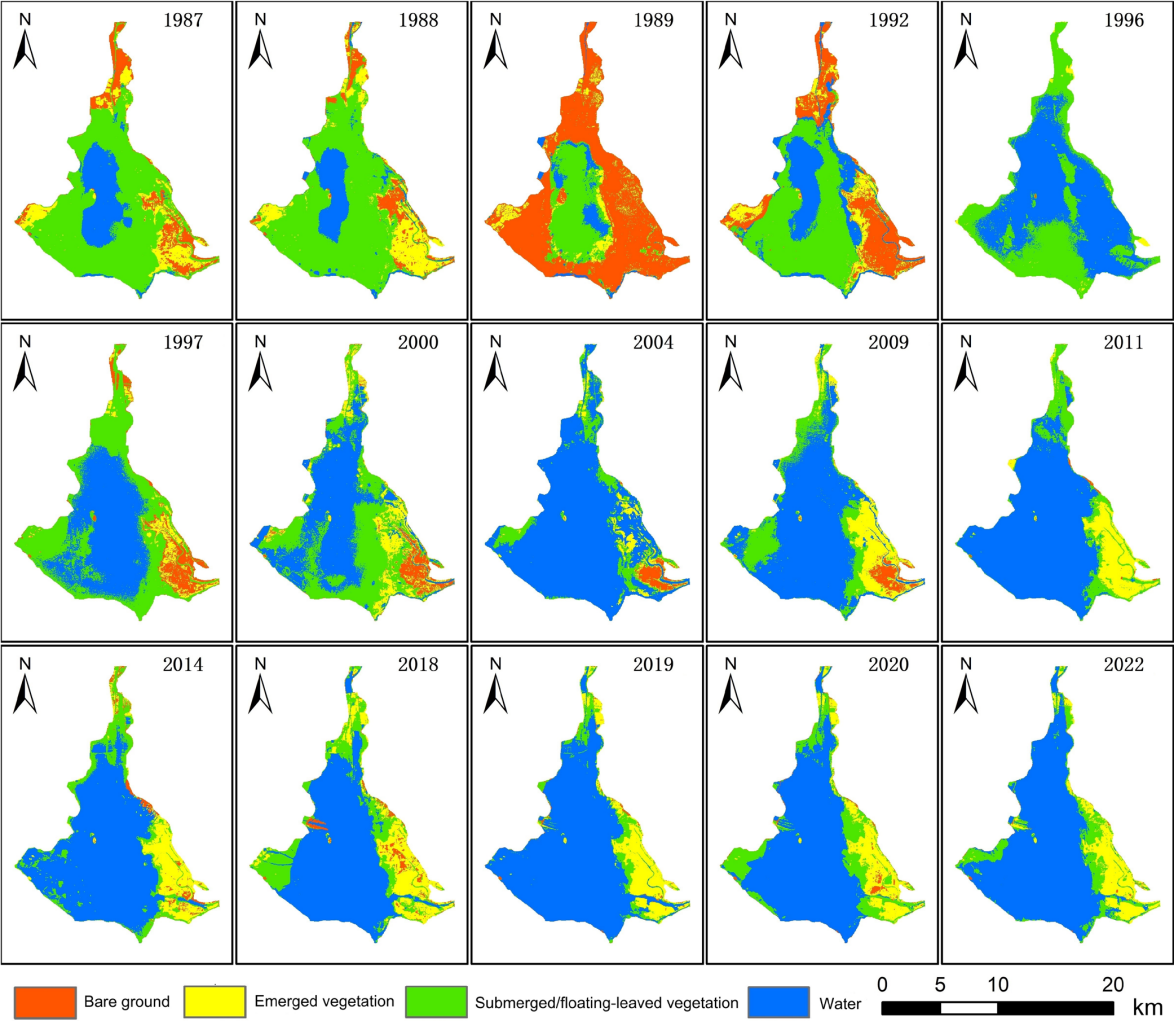
Distribution of aquatic vegetation in Dongping Lake during the summer.

By integrating historical survey records (Table 3) and remote sensing data (Figures 3 and 4), we reveal the succession of aquatic vegetation in Dongping Lake over the past four decades. The results demonstrate significant shifts in the lake's aquatic vegetation community, which can be divided into three distinct phases: the period prior to the mid‐1980s, from the mid‐1980s to the mid‐2010s, and the post‐mid‐2010s period.

**TABLE 3:** Succession of aquatic plant species in Dongping Lake (Hou et al. 2022; Pang 1999; Tian et al. 2018; Wang 1984; Yu et al. 2005).

Year	Aquatic vegetation species				
	Emergent plants	Submerged plants	Floating‐leaved plants	Free‐drifting plants	Total
1979	21	11	4	4	40
1994	6	9	4	0	19
2003–2004	4	8	3	3	18
2016	2	2	2	0	6
2020	2	2	2	0	6

#### 4.1.1 Stage 1 (Pristine Stage)

Before the mid‐1980s, historical field surveys indicate that Dongping Lake supported rich aquatic vegetation with high species diversity, vigorous growth, and substantial biomass. In 1979, the lake hosted 40 aquatic plant species, distributed in a ring‐like pattern with 85% coverage and a total biomass of 300,600 tons. Submerged vegetation was dominated by *Hydrilla verticillata* (39.60% of total biomass), *Potamogeton malaianus*, *Vallisneria natans* , and *Ceratophyllum demersum* . Floating‐leaved plants such as *Euryale ferox* , *Trapa* spp., and *Nymphoides peltata* were widely distributed, while emergent vegetation maintained notable populations of *Phragmites australis* , *Zizania latifolia* , *Typha* spp., and *Nelumbo nucifera* (Wang 1984). During this period, minimal human disturbance allowed the aquatic ecosystem to remain pristine, fostering harmonious coexistence of diverse species. Dominant taxa included *Hydrilla verticillata* , *Ceratophyllum demersum* , *Potamogeton malaianus*, *Phragmites australis* , and *Euryale ferox* .

#### 4.1.2 Stage 2 (Degradation Stage)

**FIGURE 5:**
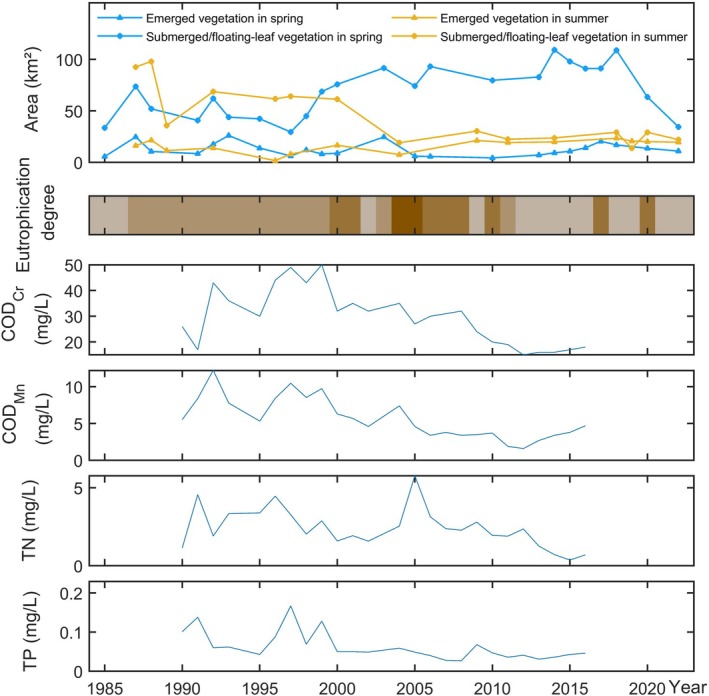
Temporal changes in aquatic vegetation area and water quality parameters.

From the mid‐1980s to the early 2000s, aquatic vegetation in Dongping Lake experienced drastic species loss, with total diversity dropping by over 50% compared to 1979. By 1994, only 19 species remained: 6 emergent, 9 submerged, and 4 floating‐leaved plants, while free‐drifting species vanished entirely (Table 3). Emergent species such as *Zizania latifolia* , *Schoenoplectus tabernaemontani* , and *Roegneria kamoji* disappeared, and *Phragmites australis* coverage shrank to 2.2 km^2^. Floating‐leaved vegetation, including *Trapa* spp. and *Euryale ferox* , declined from 70 km^2^ in 1983 to 36 km^2^ (Hou et al. 2022; Pang 1999). Remote sensing analysis corroborated this decline, showing gradual reductions in spring and summer vegetation coverage post‐1985. Spring submerged/floating‐leaved vegetation peaked at 73.7 km^2^ in 1987 but plummeted to 29.5 km^2^ by 1997, while summer coverage decreased from 90 km^2^ in 1988 to around 60 km^2^ (Figure 5).

The period from 2000 to 2014 marked a critical shift toward community homogenization. Despite ongoing species loss (Tian et al. 2018), spring submerged vegetation area slowly increased (Figure 3). This paradox arose from intensified human activities driving persistent eutrophication, which favored pollution‐tolerant species like *Potamogeton crispus* while displacing sensitive taxa. Field surveys and remote sensing data (Tian et al. 2018; Figure 3) confirm that *Potamogeton crispus* proliferated post‐2000, dominating spring submerged vegetation and peaking at over 100 km^2^ by 2015. However, summer submerged/floating‐leaved vegetation dwindled to approximately 30 km^2^ (Figure 5). Additionally, emergent vegetation in the southwestern lake vanished entirely after the 1990s, replaced by submerged/floating‐leaved species (Figure 4). Thus, from 1985 to 2015, aquatic vegetation in Dongping Lake underwent severe species depletion and structural simplification, with *Potamogeton crispus* emerging as a monoculture during spring peaks.

#### 4.1.3 Stage 3 (Stabilized Simplification Stage)

Since the mid‐2010s, the simplified vegetation structure has stabilized, with species diversity remaining low. Remote sensing reveals a sharp decline in spring submerged/floating‐leaved vegetation, dropping from 108.8 km^2^ in 2018 to 34.5 km^2^ in 2022 (Figure 5), indicating effective control of *Potamogeton crispus* proliferation. However, summer vegetation continues to decline slowly without recovery (Figure 4), suggesting no resurgence of historical dominants like *Ceratophyllum demersum* , *Potamogeton malaianus*, or *Hydrilla verticillata* . Field surveys in 2016 and 2020 identified only two submerged species: *Potamogeton crispus* (still dominant and lake‐wide) and sparsely distributed *Myriophyllum spicatum* . Floating‐leaved plants like *Nymphoides peltata* and *Trapa* spp. persist in limited areas, while emergent species such as *Zizania latifolia* and *Phragmites australis* cling to shorelines (Hou et al. 2022; Tian et al. 2018). These findings underscore the enduring simplicity of Dongping Lake's aquatic vegetation community.

### 4.2 Key Drivers of Abrupt Ecological Change

#### 4.2.1 Changes in Water Quality

Figure 5 reveals that Dongping Lake's water quality underwent a dynamic shift from deterioration to partial recovery since the 1980s (Li et al. 2018). During the early to mid‐1980s, water quality remained favorable, supporting diverse aquatic vegetation communities with high biomass. Dominant species included pollution‐sensitive submerged plants such as *Hydrilla verticillata* , *Vallisneria natans* , and *Potamogeton malaianus* (Wang 1984; Yu et al. 2005). However, water quality began declining in the late 1980s, threatening the aquatic ecosystem. By the 1990s, severe degradation pushed most areas of the lake into mesotrophic‐eutrophic states, devastating the vegetation. By 1994, aquatic plant species had decreased by over 50% compared to 1979; biomass plummeted, pollution‐sensitive species vanished, and submerged/floating‐leaved vegetation areas in both spring and summer declined steadily, reflecting the inhibitory effects of worsening pollution.

By the late 1990s, some water quality indicators improved, yet eutrophication intensified. During this period, spring submerged/floating‐leaved vegetation area reversed its decline and began expanding rapidly, while summer coverage sharply decreased (Figure 5). This paradox arose because the pollution‐tolerant species *Potamogeton crispus* thrived under elevated nutrient levels, particularly in spring (March–May) when water temperatures (14°C–21°C) aligned with its optimal growth range (Liang et al. 2019). *Potamogeton crispus* outcompeted other species, dominating the lake. However, its decay in late spring released massive nutrients, triggering secondary pollution and physicochemical changes that further degraded water quality and suppressed summer vegetation growth (Deng et al. 2020). Post‐2000, despite marked improvements in most water quality parameters, persistent eutrophication continued to drive *Potamogeton crispus* expansion and the decline of other species. Similar findings in Lake Taihu's eastern region highlight that increased ammonia, nitrogen, and phosphorus concentrations significantly inhibit aquatic vegetation, with eutrophication playing a more significant role in community degradation (Zhang et al. 2016). Thus, eutrophication served as the primary driver of Dongping Lake's vegetation decline during the 2000s.

#### 4.2.2 Water‐Level Fluctuations

Both natural and anthropogenic water level fluctuations significantly impact lake ecological management and serve as critical ecological drivers of aquatic plant growth and reproduction (Wang et al. 2019). Water level fluctuations alter nutrient dynamics, modulate interactions between macrophyte coverage, sediment resuspension‐deposition cycles, and light availability in the water column, making them a key factor in aquatic vegetation evolution. Excessively low water levels reduce lake surface area, restrict vegetation habitats, and induce drought stress, while high water levels impose light limitation on aquatic plants, hindering their growth. For Dongping Lake, emergent vegetation thrives at depths of 0.5–1.0 m, floating‐leaved vegetation at 1.0–2.0 m, and submerged vegetation cannot survive beyond 3.07 m (Yu et al. 2005). Consequently, water levels below 39.5 m or above 41 m adversely affect aquatic biota.

**FIGURE 6:**
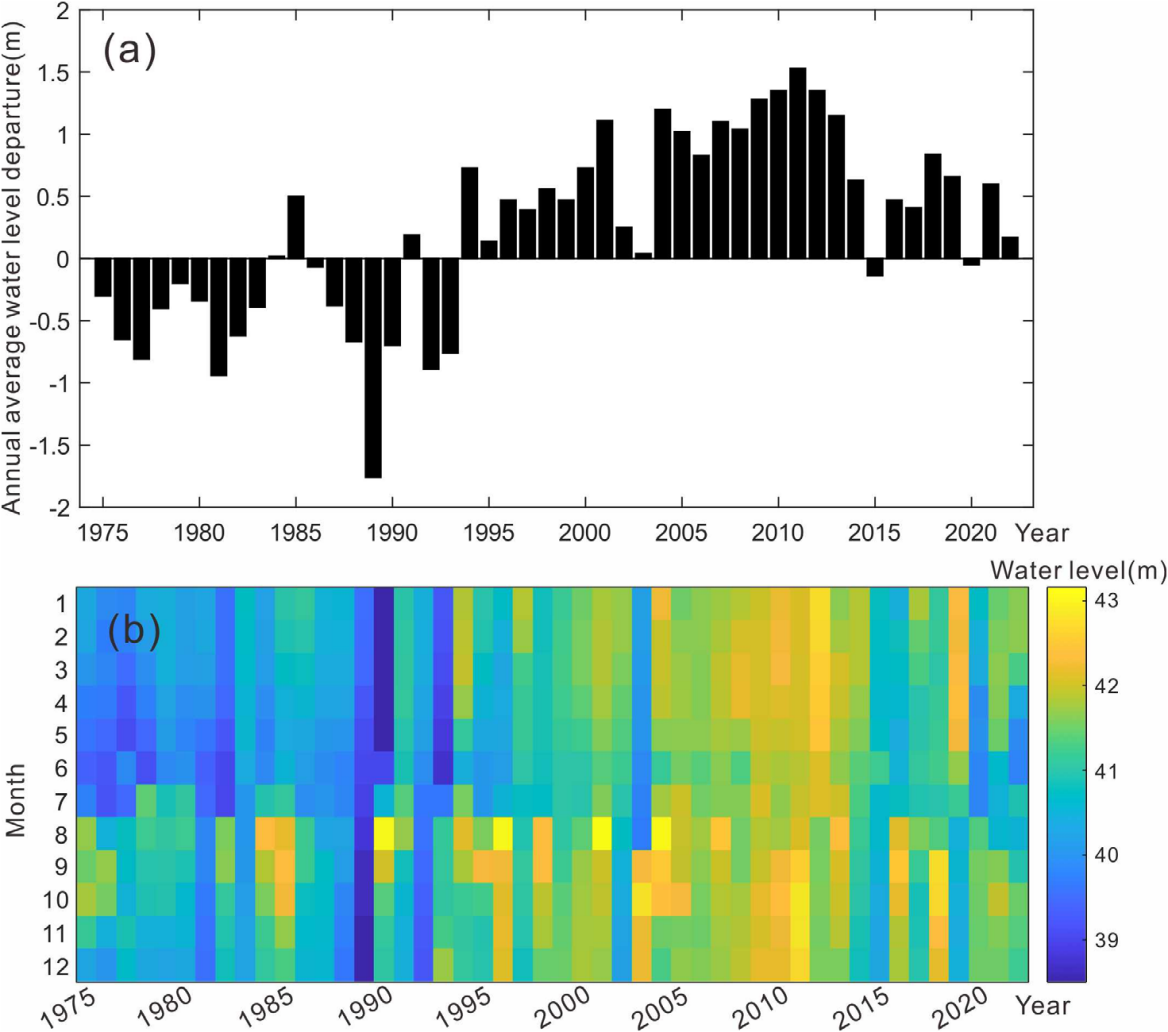
Temporal variations in water level of Dongping Lake: (a) annual anomaly from the long‐term mean and (b) monthly averages.

Since the 1970s, Dongping Lake's water level has exhibited a fluctuating upward trend (Figure 6a). During the early 1980s, annual average levels remained moderately low, creating suitable conditions for aquatic vegetation. This period saw ring‐shaped or fragmented vegetation zones with extensive coverage, high species richness, and substantial biomass. However, since the 1990s, persistent water levels exceeding 41 m (Figure 6b) have surpassed the survival threshold for emergent vegetation, leading to the disappearance of southwestern emergent communities and their replacement by submerged/floating‐leaved species. Thus, sustained high water levels have accelerated diversity loss and shifts in dominant species.

Existing studies indicate that lakes with intra‐annual water level variability support higher species richness than those with interannual fluctuations (Riis and Hawes 2002). Moderate seasonal fluctuations help maintain vegetation diversity and biomass, whereas extreme fluctuations trigger community decline. Excessively stable water levels exacerbate interspecific competition, invasive species colonization, and marshification, further undermining biodiversity (Gownaris et al. 2018). In Dongping Lake, water levels typically peak from August to October and reach their lowest between May and July (Dongping Lake Administration 2014). Since 2005, intra‐annual fluctuations have diminished, but elevated annual averages and higher spring‐early summer levels (Figure 6b) have reduced light availability during the critical germination period for aquatic vegetation, suppressing survival and reproduction. This light limitation has driven ongoing declines in species richness.

**FIGURE 7:**
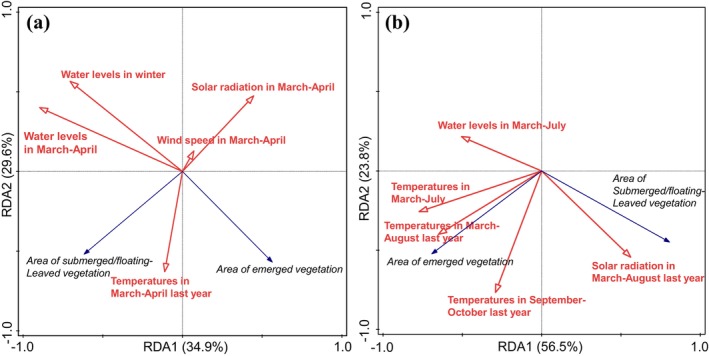
RDA ordination plots of aquatic vegetation area in spring (a) and summer (b) with environmental factors.

The RDA results (Figure 7) show that for spring aquatic vegetation, the eigenvalues of the first and second RDA axes are 0.349 and 0.296, respectively, explaining 54.1% of the adjusted variance. For summer vegetation, the eigenvalues are 0.565 and 0.238, accounting for 69.3% of the adjusted variance. The analysis reveals that water levels in March–April exhibit a negative correlation with emergent vegetation area (*p* = 0.002, contribution = 47.3%) but a positive correlation with submerged/floating‐leaved vegetation area during spring, indicating their critical role in shaping spring vegetation dynamics. Winter water levels (*p* = 0.084, contribution = 9.5%) show a weak negative impact on emergent vegetation but no significant effect on submerged/floating‐leaved vegetation.

For summer vegetation, water levels from March to July display a negative correlation with submerged/floating‐leaved vegetation area and a positive correlation with emergent vegetation area, but the effects are statistically insignificant (*p* = 0.124, contribution = 5.8%), suggesting minimal influence on summer vegetation patterns. These RDA findings corroborate qualitative analyses, confirming that reduced water level fluctuations and persistent high water levels (particularly in spring) are key drivers of aquatic vegetation degradation in Dongping Lake. Elevated spring water levels exacerbate light limitation during germination, further suppressing species recovery and reinforcing community homogenization.

#### 4.2.3 Climate Changes

Climate change exerts complex and multifaceted impacts on lake ecosystems, profoundly affecting the growth, distribution, and ecological functions of aquatic vegetation at multiple levels. Over recent decades, climatic shifts in the Dongping Lake region have been characterized by rising temperatures, reduced precipitation, and increased frequency of extreme weather events (Dongping Lake Administration 2014). RDA results indicate that solar radiation intensity significantly influences submerged/floating‐leaved vegetation (Figure 7). Radiation levels in March–April exhibit a negative correlation with submerged/floating‐leaved vegetation area in the same spring, while radiation from March to August shows a positive correlation with submerged/floating‐leaved vegetation area in the following summer. In contrast, emergent vegetation area remains largely unaffected by radiation (Figure 7). These findings suggest that elevated solar radiation may have adverse effects on the growth of *Potamogeton crispus* . Additionally, air temperature demonstrates a weak positive correlation with emergent vegetation area. While no existing studies explicitly link higher temperatures to emergent vegetation expansion, rising annual temperatures accelerate endogenous nutrient release, enhance primary productivity, and promote sustained growth of aquatic macrophytes (Zhang et al. 2020).

Furthermore, wind speed in March–April shows a negative correlation with spring submerged/floating‐leaved vegetation area (Figure 7). In shallow lakes, wind‐driven water disturbance plays a critical role in sediment resuspension and sediment–water interface dynamics, surpassing other environmental factors (Jalil et al. 2019). This process reduces water transparency and euphotic depth, hindering vegetation germination and growth. Wind‐induced waves also generate mechanical stress on plants, causing physical damage such as stem breakage and leaf detachment (Sanjeev et al. 2023).

Extreme weather events, however, trigger abrupt environmental shifts, posing challenges to water resource management (van Vliet et al. 2023) and impacting plant survival. Floods are a key disturbance factor shaping vegetation composition and diversity in floodplain lakes (Wang et al. 2019). Dongping Lake frequently experiences floods from the Dawen River and Yellow River. Historically, 45% of years in Dongping County recorded summer‐autumn floods, with six major flood events occurring between 1980 and 1990 alone (Dongping Lake Administration 2014). During floods, rapid water level rises submerge vegetation, while sediment resuspension increases suspended solids, nitrogen, and phosphorus concentrations, further reducing water clarity (Wang et al. 2019; Bornette et al. 2024). Floods also limit oxygen supply, suppress plant growth and germination, and diminish species diversity, rendering ecosystems highly vulnerable (Wang et al. 2019). For instance, heavy rainfall in the Dawen River basin in late July 1996, compounded by Yellow River floods in August, caused Dongping Lake's water storage to surge to 640 million cubic meters by August 18 (Dongping Lake Administration 2014). Remote sensing imagery from August 17 of the same year (Figure 4) reveals near‐total inundation of the Dawen River wetland, significant loss of emergent vegetation, and disruption of remaining vegetation patterns.

#### 4.2.4 Human Disturbances

As a regulating reservoir for the eastern route of China's SNWDP, Dongping Lake began Phase I construction in 2002 and commenced full operation in November 2013, with a designed flow rate of 100 m^3^/s (He et al. 2021). During water diversion periods, flow velocities in Dongping Lake remain sluggish (mostly below 0.04 m/s), while water levels are maintained between 40.5 m and 42.5 m (He et al. 2014), resulting in elevated and stable lake levels. This artificial regulation has imposed persistent adverse impacts on aquatic vegetation: expanded water surfaces and abrupt habitat shifts have driven community homogenization (Zhou 2022). Conversely, stable water levels mitigate drought‐induced lake shrinkage and plant desiccation, partially preserving vegetation area and biomass.

The project's influence extends to water quality. Its stringent water quality requirements have accelerated pollution control in Dongping Lake and the Dawen River basin (Yu et al. 2005). Clean water inflows during diversion shorten the water renewal cycle, increase storage capacity, and enhance pollutant dilution while curbing endogenous nutrient accumulation (Sun et al. 2024). However, diversion also risks sediment disturbance, reducing transparency and releasing nutrients (Sun et al. 2024). Post‐implementation, Dongping Lake's water renewal cycle shortened dramatically from 337 days to 23 days, creating dynamic flow conditions. This rapid alteration in nutrient distribution and resuspension of endogenous pollutants modified suspended sediment loads and transparency, influencing eutrophication and algal dynamics (He et al. 2021). For instance, emergency diversions from April to June 2019 significantly elevated suspended particulate matter concentrations, likely contributing to the historically low submerged/floating‐leaved vegetation area recorded in August that year. Additionally, nutrient inputs from diverted external water sources may exacerbate eutrophication, raising cyanobacterial bloom risks during summer and autumn—a major challenge for ecological regulation. Overall, water quality in Dongping Lake has markedly improved since the project's implementation, fostering partial vegetation recovery. However, persistent nutrient accumulation and delayed remediation hinder comprehensive eutrophication control, underscoring ongoing ecological management challenges.

### 4.3 Implications for Lake Restoration and Management

The above research reveals that over the past 40 years, the aquatic vegetation in Dongping Lake has undergone significant degradation due to poor water quality, high water levels, low hydrological fluctuations, and intensified human activities. This degradation has not only impacted the socio‐economic functions of the lake but also harmed its ecological services.

The drastic decline in aquatic plant species richness—from 40 species in 1979 to only 6 in recent surveys—reflects a severe erosion of biodiversity in Dongping Lake. This loss extends beyond mere numbers; it undermines ecosystem stability, reduces functional redundancy, and diminishes the lake's capacity to provide critical services such as water purification, habitat provisioning, and nutrient cycling. Globally, biodiversity loss is recognized as a key driver of ecosystem degradation, with cascading effects on human health and livelihoods (Simmonds et al. 2023). Similar patterns of species impoverishment have been documented in other aquatic and terrestrial systems, where declining populations of keystone or sensitive species signal broader environmental stress (Kumar et al. 2025). In Dongping Lake, the disappearance of pollution‐sensitive submerged species and the homogenization of the community toward a monoculture of *Potamogeton crispus* exemplify how eutrophication and hydrological alterations can simplify ecosystems and reduce their resilience to future disturbances.

Therefore, protecting and restoring aquatic vegetation communities is crucial for the sustainable development of Dongping Lake and its surrounding areas. Our analyses identify water pollution and eutrophication as primary drivers of declining species richness and community simplification in Dongping Lake's aquatic vegetation over four decades. Water quality improvements correlate strongly with enhanced watershed management and reduced pollutant influx. Thus, it is imperative to strengthen watershed pollution control, strictly regulate pollutant inputs into the lake, minimize excessive nutrient and organic loads from agricultural areas, aquaculture, and upstream regions, and prevent sediment overload from deteriorating water quality—all of which are vital for aquatic vegetation growth (Zhang et al. 2017). Additionally, activities such as fishing and recreational development fragment the lake area, disrupting the continuity of vegetation habitats and facilitating the influx of nutrients and pollutants. Restricting these activities can improve water transparency and mitigate eutrophication.

Water level fluctuations significantly influence aquatic vegetation biomass and spatial distribution in Dongping Lake. Effective lake management and restoration must therefore prioritize hydrological regulation. The SNWDP has substantially altered Dongping Lake's natural water level dynamics through artificial control. To balance water diversion needs, flood control, and ecological health, we recommend maintaining water levels within 39.5–41.0 m. Besides, intentional seasonal fluctuations should be introduced to enhance the primary productivity of the ecosystem (Gownaris et al. 2018).

The restoration of aquatic vegetation in eutrophic lakes is a global ecological challenge. Over recent decades, significant efforts and financial resources have been devoted to testing various restoration measures, yet the outcomes often fall short of expectations. Therefore, it is crucial to systematically restore species diversity, biomass, and distribution areas of aquatic vegetation based on the unique characteristics of different plant species. The recovery of submerged macrophytes is pivotal for rehabilitating eutrophic waters and maintaining aquatic ecosystem health. These plants demonstrate advantages in nutrient uptake, alleviate eutrophication through allelopathic effects that suppress algal growth, and inhibit phytoplankton proliferation. Consequently, artificial cultivation of submerged species like *Myriophyllum spicatum* and *Ceratophyllum demersum* can enhance dominant species populations. For pollution‐tolerant species such as *Potamogeton crispus* , mechanical harvesting and the release of herbivorous fish can be employed to control its growth scale (Hou et al. 2022). Simultaneously, emergent and floating‐leaved plants like *Trapa* spp., *Euryale ferox* , and *Zizania latifolia* should be reintroduced through artificial planting in shallow areas of Dongping Lake to comprehensively rehabilitate the aquatic vegetation community.

## 5 Conclusions

This study reconstructs the long‐term dynamics of aquatic vegetation in Dongping Lake over the past 40 years by integrating remote sensing monitoring (Landsat imagery analyzed via SVM classification) with historical field survey data. RDA was employed to assess the relationships between vegetation changes and key environmental drivers. The main findings are:

1. The aquatic vegetation community in Dongping Lake has undergone three stages of progressive degradation succession. Before the mid‐1980s (pristine stage), 40 plant species were recorded in 1979, with a coverage rate of 85% and a biomass of 300,600 tons. Submerged, floating‐leaved, and emergent plants formed a stable, complex community distributed in a ring‐like pattern. From the mid‐1980s to the mid‐2010s (degradation stage), species decreased by 50% (only 19 species remained by 1994), and free‐floating plants disappeared completely. Remote sensing showed that the area of spring submerged/floating‐leaved vegetation sharply declined from 73.7 km^2^ in 1987 to 29.5 km^2^ in 1997. After 2000, an ecological transition occurred: the pollution‐tolerant species *Potamogeton crispus* formed a spring monoculture (exceeding 100 km^2^ by 2015), while summer vegetation continued to decline. Since the mid‐2010s (stabilized simplification stage), the area of spring submerged/floating‐leaved vegetation plummeted from 108.8 km^2^ in 2018 to 34.5 km^2^ in 2022. Currently, only two submerged species remain: *Potamogeton crispus* (dominant throughout the lake) and sparsely distributed *Myriophyllum spicatum* . Floating‐leaved and emergent plants are confined to scattered shoreline areas, indicating intensified community homogenization and ongoing loss of ecological resilience.
2. The RDA and temporal trend analysis within this study directly identify water quality parameters and water level fluctuations as primary factors associated with aquatic vegetation degradation in Dongping Lake. Deteriorating water quality (particularly eutrophication) and persistently high water levels, especially during the spring germination period, show significant negative correlations with vegetation area and are linked to community homogenization. Meteorological factors (solar radiation, wind speed) also demonstrate measurable correlations with vegetation coverage in our analysis.
3. The impacts of human activities, notably aquaculture operations and the operation of the SNWDP, are primarily indirect, mediated through their modification of the lake's physicochemical environment. Our analysis of hydrological data shows that SNWDP operations have led to elevated and stabilized water levels, a regime that exacerbates light limitation. While the project's water quality requirements have contributed to improved pollutant control in recent years, its operational pattern has concurrently altered the natural hydrological rhythm, creating conditions unfavorable for diverse vegetation recovery.
4. The findings provide a targeted scientific basis for ecological restoration and adaptive management of Dongping Lake. To reverse ecosystem degradation of this vital floodplain lake ecosystem, an integrated management strategy is essential: (i) implementing watershed‐scale pollution control to reduce nutrient inputs and improve water quality; (ii) restoring ecological hydrological regimes by optimizing water levels and reintroducing seasonal fluctuations; (iii) initiating active vegetation restoration, including reintroducing native submerged species, controlling *Potamogeton crispus* dominance, and rehabilitating emergent and floating‐leaved communities; and (iv) establishing long‐term monitoring and adaptive management to evaluate and refine interventions.

This study demonstrates the effectiveness of integrating remote sensing, field survey, and environmental monitoring data to reconstruct long‐term ecological dynamics in floodplain lakes. The directly observed vegetation succession patterns and quantitatively identified drivers provide a robust scientific basis for the ecological restoration of Dongping Lake and offer valuable insights for managing similar eutrophic freshwater ecosystems globally. Future research should focus on long‐term in situ monitoring of vegetation recovery and the interactive effects of multiple drivers under global climate change to refine adaptive management strategies.

## Author Contributions

**Yingying Chen:** conceptualization (lead), data curation (lead), formal analysis (lead), funding acquisition (lead), investigation (lead), methodology (lead), project administration (lead), resources (lead), software (lead), supervision (lead), validation (lead), visualization (lead), writing – original draft (lead), writing – review and editing (lead). **Yanyu Ji:** data curation (supporting), formal analysis (supporting), investigation (supporting), software (supporting), visualization (supporting), writing – review and editing (supporting). **Qinghui Zhang:** data curation (supporting), investigation (supporting), resources (supporting), writing – review and editing (supporting). **Yunwei Zhang:** investigation (supporting), resources (supporting), writing – review and editing (supporting). **Shi‐Yong Yu:** funding acquisition (supporting), resources (supporting), writing – review and editing (supporting). **Shiyue Chen:** conceptualization (supporting), funding acquisition (supporting), resources (supporting), writing – review and editing (supporting).

## Conflicts of Interest

The authors declare no conflicts of interest.

## Data Availability

The data that support the findings of this study are openly available in the Supporting Information.
